# Dietary soybean protein ameliorates high-fat diet-induced obesity by modifying the gut microbiota-dependent biotransformation of bile acids

**DOI:** 10.1371/journal.pone.0202083

**Published:** 2018-08-13

**Authors:** Keita Watanabe, Miki Igarashi, Xuan Li, Akiho Nakatani, Junki Miyamoto, Yuka Inaba, Asuka Sutou, Tsutomu Saito, Takumi Sato, Nobuhiko Tachibana, Hiroshi Inoue, Ikuo Kimura

**Affiliations:** 1 Department of Applied Biological Science, Graduate School of Agriculture, Tokyo University of Agriculture and Technology, Fuchu, Tokyo, Japan; 2 AMED-CREST, Japan Agency for Medical Research and Development, Chiyoda-ku, Tokyo, Japan; 3 Metabolism and Nutrition Research Unit, Institute for Frontier Science Initiative, Kanazawa University, Takara-machi, Kanazawa, Ishikawa, Japan; 4 Health and Nutrition Research Department, Research and Development Division for Future Creation, Fuji Oil Co., Ltd., Tsukuba-mirai, Ibaraki, Japan; Tokyo University of Agriculture, JAPAN

## Abstract

The consumption of soybean protein has well-known favorable metabolic effects (e.g., reduced body weight, body fat, hyperglycemia, insulin resistance, hepatic steatosis, and lipogenesis). These effects of soy protein have been linked to modulation by the gut microbiota; however, the dynamic interplay among these factors remains unclear. Accordingly, we examined the metabolic phenotype, intestinal BA pool, and the gut microbiome of male C57BL/6 mice that were randomized to receive either a regular high-fat diet (HFD) or HFD that contained soybean protein isolate (SPI) in place of dairy protein. The intake of SPI significantly reduced the HFD-induced weight gain and adipose tissue mass accumulation and attenuated hepatic steatosis. Along with an enhancement in the secretion of intestinal Glucagon-like peptide-1 (GLP-1), an enlarged cecal BA pool with an elevated secondary/primary BA ratio was observed in the mice that consumed SPI, while fecal BA excretion remained unaltered. SPI also elicited dramatic changes in the gut microbiome, characterized by an expansion of taxa that may be involved in the biotransformation of BAs. The observed effects were abolished in germ-free (GF) mice, indicating that they were dependent on the microbiota. These findings collectively indicate that the metabolic benefits of SPI under the HFD regime may arise from a microbiota-driven increase in BA transformation and increase in GLP-1 secretion.

## Introduction

In 2016, an estimated 1.9 billion adults and 340 million children and adolescents worldwide were overweight or obese [1]. The current obesity epidemic is accompanied by an increased incidence and prevalence of cardiometabolic diseases, such as type II diabetes mellitus (T2DM) and hepatic steatosis, and several cancers that affect humans of all ages. The etiology of obesity is thought to involve a complex interplay between genetic susceptibility and demographic and lifestyle factors. Dietary modification, in the context of lifestyle changes, is regarded as an essential component of strategies for the prevention and treatment of obesity and related conditions.

A growing body of evidence suggests that the pathophysiology of metabolic diseases is associated with dysbiosis of the gut microbiota and its reciprocal interaction with the pool size and composition of bile acids (BAs) [2, 3]. In addition to being important mediators of lipid absorption, BAs also act as hormone-like regulators of energy expenditure, inflammation, lipids, carbohydrates, and their own metabolism by acting predominantly in enterohepatic and other peripheral organs [4, 5]. For instance, in enteroendocrine L cells, BAs regulate the production and secretion of Glucagon-like peptide-1 (GLP-1) via opposite effects on Takeda G protein-coupled receptor 5 (TGR5) and farnesoid X receptor (FXR) [6–8]. The activation of TGR5, the expression of which parallels L-cell density along the gastrointestinal tract with maximal expression in the colon, induces preproglucagon gene expression and GLP-1 secretion [7]. TGR5 activation in pancreatic α-cells induces pro-convertase-1 expression, shifting glucagon production to GLP-1, thereby increasing β-cell mass and function in a paracrine manner [6]. By contrast, FXR activation represses preproglucagon gene expression and GLP-1 secretion in the ileum by inhibiting glycolysis and carbohydrate-responsive element-binding protein activity in L cells [8, 9]. BAs can alter intestinal microbiome, because BAs exert antimicrobial activity through their detergent action and, conversely, augment the growth of some bacterial strains in their free or conjugated forms [10, 11]. The microbiota modifies the BA pool by metabolizing the primary BAs synthesized in hepatocytes into their secondary forms in the small intestine via a number of reactions, including deconjugation, dehydrogenation, dihydroxylation, and epimerization. The BAs that escape reabsorption by enterocytes in the distal ileum are either further deconjugated by the colonic microbiota and passively absorbed in the colon or lost via the feces [12]. A dysbiosis associated with increases in BA synthesis, fecal primary BAs, and the primary to secondary BA ratio has been observed in patients with nonalcoholic steatohepatitis [13, 14]. Additionally, a rapidly enlarged intestinal pool of BAs and the consequent compositional alterations in the gut microbiota frequently accompany the consumption of a high-fat diet (HFD) [15]. Taken together, the gut microbiota indeed modulates the potency of the physiological functions of BAs and is reciprocally modulated by BA metabolism.

Soy protein is considered a complete protein, i.e., it contains most of the essential amino acids that are found in animal proteins. The nutritional value of soy protein is roughly equivalent to that of high-value animal protein. Soy protein isolate (SPI) includes 20% β-conglycinin [16], which has well-documented favorable metabolic effects (e.g., it reduces body weight, body fat, hyperglycemia, insulin resistance, hepatic steatosis, and lipogenesis) according to a number of animal and human studies [16–21]. These effects of soy protein have been linked to alterations in BA metabolism [13–15], which is subject to modulation by the gut microbiota [12, 22]; however, the dynamic interplay among these factors remains unclear. In the present study, we hypothesized that the intake of SPI would exert protective effects against weight gain and obesity resulting from an HFD regime by modulating the gut microbiota and BA signaling. Accordingly, we investigated changes in the size and composition of the BA pool and the cecal microbiome using an HFD-induced obesity mouse model to establish the correlation between soy protein intake, the gut microbiome, and BA signaling and to provide insight into the tailored application of soy protein as a dietary intervention for weight management.

## Results

### Intake of SPI reduced HFD-induced weight gain and hepatic steatosis and enhanced intestinal GLP-1 secretion

**Fig 1:**
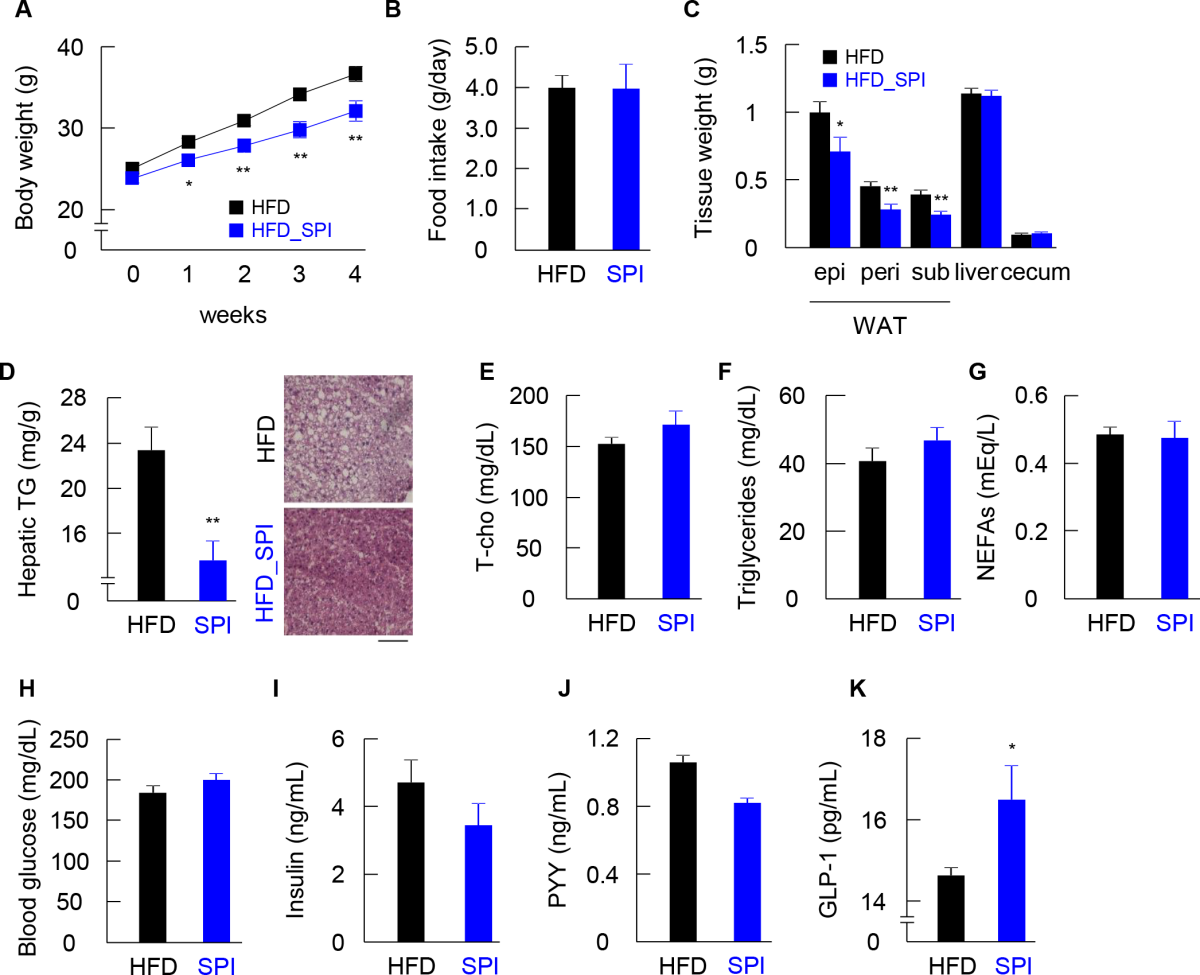
Phenotypic and histological changes in HFD and HFD-SPI-fed mice. CONV-R mice were characterized for body weight gain (A), food intake (B), weight of epididymal, perirenal, subcutaneous adipose tissues and liver and cecum (C), hepatic triglycerides and histology of hepatocytes by H&E staining (D) (n = 9–10). Plasma total cholesterol (E), plasma triglyceride (F), plasma NFFA (G), blood glucose (H), plasma insulin (I), plasma PYY (J), and plasma GLP-1 (K) (n = 8–10). Data are expressed as means ± SE. Differences were assessed by Student's t-test. Significance is established at adjusted *p* < 0.05 (* *p* < 0.05, ** *p* < 0.01).

Body weights of conventionally raised (CONV-R) and GF mice fed an HFD or HFD-SPI were monitored weekly following the initiation of the diet treatment, and the tissue characteristics and biochemical properties of plasma were assessed at the end of the treatment period. Remarkably, the HFD-SPI mice began to exhibit less weight gain than that in HFD mice immediately after 1 week of treatment, and the difference in body weights between groups increased over time (Fig 1A). To note, SPI did not affect body weight in the mice fed a low-fat diet (S1 Fig). There was no difference in food intake between HF and HFD-SPI groups (Fig 1B). The weights of epididymal, perirenal and subcutaneous adipose tissues were also significantly lower in HFD-SPI mice than in HFD mice, whereas no significant differences were observed in the liver and cecum between groups (Fig 1C). Dietary SPI supplementation led to a significant reduction in hepatic triglyceride accumulation (Fig 1D). Consistent with the measurements, hematoxylin and eosin-stained hepatic sections from HFD-SPI mice exhibited a reduction in vacuolated hepatocytes, indicating a reduced degree of steatosis, compared to that of the HFD group (Fig 1D). Plasma levels of total cholesterol, triglycerides, non-esterified fatty acid, and glucose did not differ between treatments (Fig 1E–1H). Although plasma levels of insulin and the anorexigenic gut hormone peptide YY (PYY) were similar in HDF and HFD-SPI fed mice (Fig 1I and 1J), plasma levels of GLP-1 were greatly enhanced in response to the HFD-SPI intervention (Fig 1K).

### Beneficial metabolic effects of SPI were abolished under GF conditions

**Fig 2:**
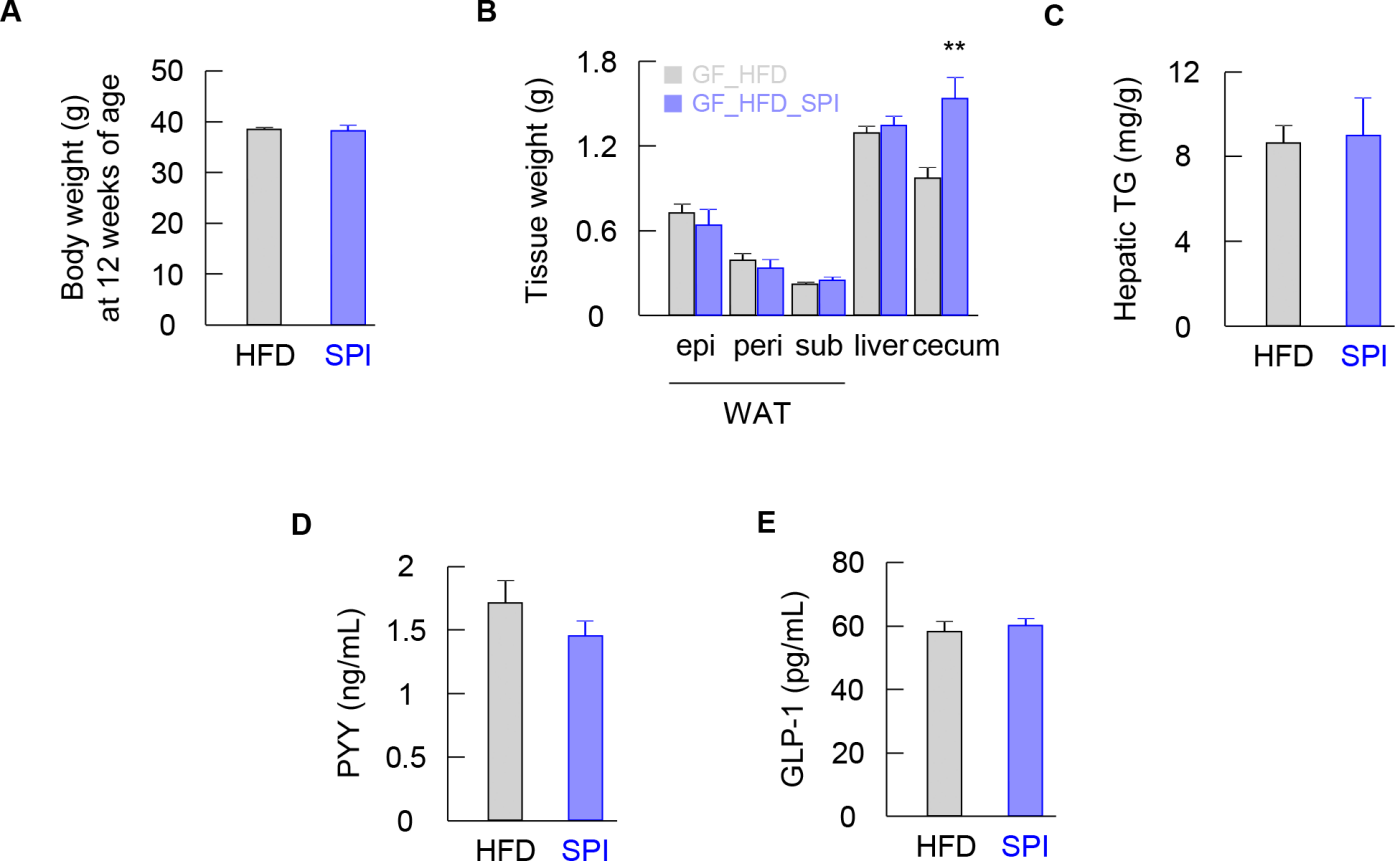
Comparison between HFD and HFD-SPI-fed mice under GF condition. GF mice were characterized for body weight at 12 weeks of age (A), weight of epididymal, perirenal, subcutaneous adipose tissues and liver and cecum (B), and hepatic triglyceride (C) (n = 8). Plasma PYY (D) and GLP-1 (E) were measured by ELISA (n = 8). Data are expressed as means ± SE. Differences were assessed by Student's t-test. Significance is established at adjusted *p* < 0.05 (** *p* < 0.01).

Concomitantly, to verify whether the beneficial metabolic effects associated with the intake of SPI depend on the gut microbiota, the body weights of GF mice fed an HFD or HFD-SPI for 4 weeks were measured. In GF mice, the protective effects of SPI against fat accumulation in adipose tissues and hepatic steatosis were not observed (Fig 2A–2C). In addition, although changes in PYY plasma levels were similar in CONV-R and GF mice, the effects on plasma GLP-1 levels were abolished (Fig 2D and 2E). Thus, the protective effects against hepatic steatosis and promotion of GLP-1 secretion were abolished under GF conditions.

### SPI supplementation in HFD altered fecal and cecal BA profiles

**Fig 3:**
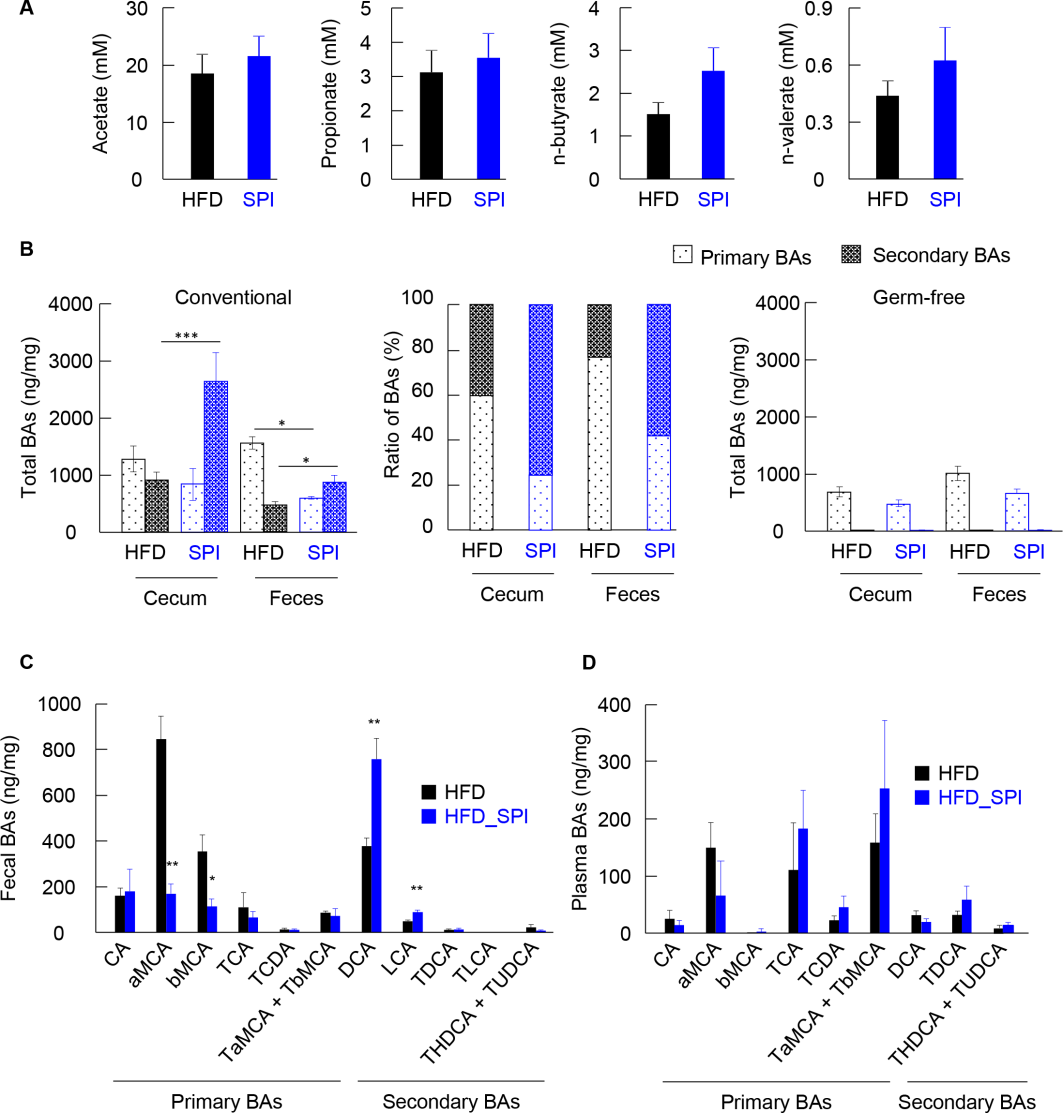
SCFAs and BAs profiles in HFD and HFD-SPI-fed mice. Fecal SCFAs were measured in HFD- and HFD-SPI-fed CONV-R mice (A). BAs were determined in cecal contents and feces of CONV-R and GF mice (B and C). The data were shown in the absolute amounts (B, left and right) and ratio (B, middle) of primary and secondary BAs, and individual BAs (C). Individual BAs were determined in plasma of CONV-R mice (D). Data are expressed as mean ± SE (n = 7–10). Differences were assessed by Student's t-test. Significance is established at adjusted *p* < 0.05 (* *p* < 0.05, ** *p* < 0.01).

It is well known that short-chain fatty acids (SCFAs) and BAs are major gut microbiota metabolites related to gut microbiota-mediated GLP-1 secretion [23–25]. Hence, SCFA concentrations in the feces of mice fed an HFD and HFD-SPI were quantified. Fecal short chain fatty acids were not different between two groups (Fig 3A). Next, to assess the effects of SPI under an HFD regime on the synthesis, transformation, and metabolic fate of BAs, their concentrations in the cecal content and feces of mice fed an HFD and HFD-SPI were quantified. Overall, the cecal levels of secondary BAs were significantly increased in response to an HFD-SPI diet with a moderate decrease in the concentration of primary BAs, contributing to an enlarged pool of BAs (defined as the sum of primary and secondary BAs) and a drastically elevated secondary to primary BA ratio (Fig 3B left and middle). Notably, although the HFD-SPI mice were associated with a much higher cecal BA concentration, they exhibited lower total fecal excretion of BAs than that of the HFD-fed mice (Fig 3B, left). These results indicate that the BAs, especially the secondary BAs, were absorbed in the colon in the HFD-SPI mice to a significant extent. Particularly, in the feces, levels of secondary BAs, including deoxycholic acid (DCA) and lithocholic acid (LCA), were significantly greater in the HFD-SPI mice than in the HFD controls (Fig 3C). In the context of primary BAs, both α-muricholic acid, the most abundant primary BA species in rodents, and β-muricholic acid were greatly reduced in HFD-SPI mice compared to the HFD controls (Fig 3C). In contrast, there was no significant difference in plasma individual BAs between HFD and HFD-SPI groups (Fig 3D). On the other hand, the cecal and fecal BA pool contained few secondary BAs in GF mice, as expected (Fig 3B Right). Additionally, there was no significant difference in primary BAs of cecal contents and feces between groups. These results collectively suggest an overall promotion of the intestinal microbiota-driven transformation of primary BAs towards their secondary forms in response to the HFD-SPI diet, and that a BA pool rich in secondary species favored the reabsorption of BAs in the colon.

### Intake of SPI modified the HFD-associated gut microbiota

**Fig 4:**
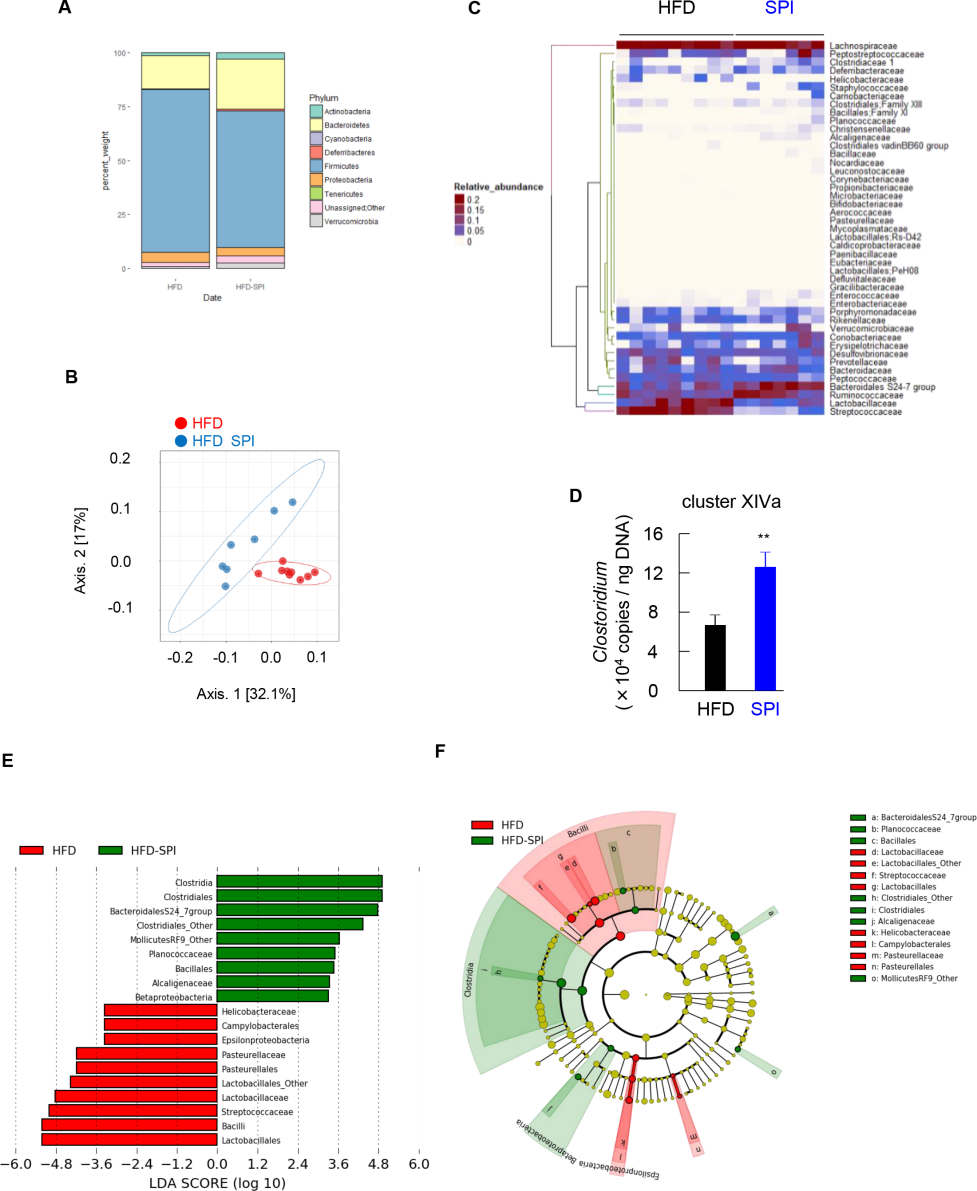
Changes in the fecal microbiota in the HFD-SPI-fed mice compared to the HFD controls. (A) Relative abundance of major taxonomic groups at phylum level, (B) principle coordinate analysis (PCoA), and (C) heatmap of relative abundance of major taxonomic groups at family level (mean relative abundance > 0.1%) of the fecal microbiota in HFD-SPI-fed mice vs. the HFD controls based on unweighted Unifrac distances between diet groups. (D) Clostridium cluster XIVa of the fecal microbiota was measured by quantitative real-time PCR. Data are expressed as mean ± SE (n = 6–8). Differences were assessed by Student's t-test. Differences were assessed by Student's t-test. Significance is established at adjusted *p* < 0.05 (** *p* < 0.01). Linear discriminative analysis (LDA) effect size (LEfSe) analyses of statistically significant taxa at family level. Taxa were sorted by degree of difference (E) and overlaid on a taxonomic cladogram (F). Only the taxa meeting a significant LDA threshold value of >2.0 are shown. n = 9 for HFD group, n = 7 for HFD-SPI group.

A taxonomic analysis of the fecal microbiota showed that the bacterial population associated with either diet group was dominated at the phylum level by Firmicutes (58.05% for HFD and 55.98% for HFD-SPI) and Bacteroidetes (30.75% for HFD and 29.93% for HFD-SPI) followed by Proteobacteria (6.75% for HFD and 7.00% for HFD-SPI). Deferribacteres and Actinobacteria also constituted 0.58% and 0.48% of the HFD-associated microbial population, respectively, and there was a significant increase in the relative abundance of these two phyla (1.60% and 1.23%) in the HFD-SPI microbiota (Fig 4A). A principal coordinate analysis (PCoA) was employed based on unweighted UniFrac distances to elucidate the differences in taxonomic composition between diet treatments. The PCoA plot indicated significant clustering according to diet type, with a complete separation of the gut microbiota of HFD-SPI from that of HFD along the PCoA1 axis (explaining 58.2% of overall variation) (Fig 4B). Pairwise comparison by permutational multivariate analysis of variance (PERMANOVA) revealed that the differences between groups were statistically significant (P = 0.001). A heatmap summarizing the relative taxonomic abundances (Fig 4C) revealed that SPI supplementation in an HFD altered the gut microbiota at the family level. In addition, *Clostridium* cluster XIVa, which is thought to be a major producer of secondary BAs by 7α-dehydroxylation [26], was increased in the fecal microbiota of the mice fed on HFD-SPI (Fig 4D). The linear discriminant analysis (LDA) effect size (LEfSe) algorithm was applied to further identify the taxa with the greatest difference in abundance between the HFD controls and the HFD-SPI-treated mice. A bar chart (Fig 4E) and cladogram (Fig 4F) generated from LEfSe confirmed that the bacteria inhabiting the HFD and HFD-SPI mice clustered separately. In particular, at the family level, the HFD-associated microbiota was characterized by predominant increases in Streprococcaceae and Lactobacillaceae belonging to the order Lactobacillales and class Bacilli, whereas differential enrichment of Bacteroidales S24-7, Clostridiales_Other, MollicutesRF9_Other, Planococcaceae, and Alcaligenaceae was observed in the fecal microbiota of HFD-SPI mice. These results collectively indicate that replacing dairy proteins with SPI in an HFD significantly modifies the gut microbiome.

## Discussion

Soy protein has been credited with a number of metabolic benefits that are explained by diverse underlying mechanisms. In the present study, we demonstrated that replacing dairy protein in an HFD with soy protein confers resistance to HFD-induced weight gain and fat mass accumulation in multiple tissues along with the augmented secretion of GLP-1; these effects were abolished in GF conditions. The results of this study also indicated that the beneficial effects of consuming soy protein under an HFD regime are associated with an expanded cecal BA pool with an elevated secondary/primary BA ratio, without increases in fecal BA excretion. Consistent with our findings, an altered BA pool with a secondary/primary BA ratio has been observed in multiple metabolic conditions related to obesity, such as profound intestinal inflammation [27] and cirrhosis [28]. Moreover, we found that the circulating level of cholesterol associated with HFD-SPI treatment remained unaltered compared to that in the HFD controls. After synthesis and secretion, most BAs return to the enterohepatic circulation via active reabsorption in the enterocytes of the distal ileum and passive transport in the colon with the rest undergoing fecal elimination. In this regard, it has frequently been proposed that soy protein binds to BAs and prevents their reabsorption (i.e., sequestration) in the gastrointestinal tract, leading to increased fecal BA excretion and driving the hepatic uptake of plasma cholesterol for BA biosynthesis to compensate for the fecal BA loss (i.e., hypocholesterolemic effect) [29]. However, the lipid-lowering effect of SPI by reducing intestinal cholesterol absorption and increasing fecal bile acid excretion was not observed in HFD-fed mice in this study.

The SPI treatment reduced hepatic triacylglycerol in the mice fed the high-fat diet (Fig 1D), which possibly was induced by the major protein of the SPI, β-conglycinin [16]. In the previous report, dietary β-conglycinin exhibited preventive effects of fatty liver in either the mice fed high-fat diet or the OLETF rats, which might be caused by a reduction of PPARγ2, which is one of contributors of hepatic steatosis [20, 21, 30]. On the other hand, SPI did not modify plasma triacylglycerol levels in this study, as well as in the studies that used β-conglycinin (Fig 1), suggesting that the major target of SPI as well as β-conglycinin is the liver. In contrast to the animal studies, a clinical study suggested that β-conglycinin reduces serum triacylglycerol levels as well as visceral fat in human [31].

The gut microbiota is also involved in modulating the pool composition of BAs and their signaling by catalyzing the biotransformation of primary BAs to their secondary forms [32, 33]. Enzymes involved in various transformation reactions have been characterized from cultured gut bacteria and exhibit taxon-specific distributions. More recently, bioinformatics approaches have revealed greater diversity in isoforms of these enzymes and in the microbial species in which they are found [32–34]. Therefore, the distribution of resulting secondary species in the BA pool and the functional roles of BAs may be profoundly affected by the microbial community structure and function. In this study, the alteration of microbiome structure was confirmed in the mice that exhibited the increase of secondary BAs in the cecum contents and feces after SPI treatment. Since bile salt hydrolase (BSH) is widely present in Actinobacteria [32], the significant increase in the abundance of this phylum in response to SPI might contribute to the augmented biotransformation of BAs (Fig 4A). Intriguingly, SPI treatment increased one of the rRNA clusters, *Clostridium* cluster XIVa, which includes bacteria known to produce secondary BAs from primary BAs by 7α-dehydroxylation [26], in the fecal microbiota of the mice (Fig 4D). Previous analyses have shown that *Ruminococcaceae*, which is one of member in *Clostridium* cluster XIVa, is positively correlated with DCA and the ratio of DCA to CA in a rodent model of cirrhosis [28] and exhibits bile salt hydrolase (BSH) activity [33], thereby catalyzing the deconjugation of BAs, the gatekeeping reaction for further biotransformation (e.g., oxidation and dehydroxylation) of primary BAs, in the distal ileum and the proximal colon. Notably, *Ruminococcaceae* was increased in the mice by SPI treatment (Fig 4C). Therefore, we speculate that the intake of SPI in an HFD promoted the turnover of primary BA by causing the expansion of specific bacteria, which could produce secondary BAs from primary BAs. In addition, the taxonomic profile of the fecal microbiome also exhibited changes in several unclassified taxa and those of unknown relationship with BA metabolism in response to SPI (Fig 4). Since BAs and the gut microbiota reciprocally affect the composition and size of each other, increases in the relative abundance of some taxa may arise from the reduction or depletion of others. This also indicates that bacterial taxa with key roles in modulating BA metabolism could remain undescribed.

Furthermore, our results suggest that the resistance to HFD-induced weight gain conferred by SPI was probably a consequence of the enhanced secretion of intestinal GLP-1, which has essential regulatory roles in satiety, gastric emptying, and glucose tolerance by promoting β-cell survival and proliferation. Our findings agree with those of a previous report indicating that the augmented conversion of primary BAs to secondary BAs by the gut microbiota promotes the synthesis and secretion of GLP-1 via a signaling pathway mediated by TGR5, which demonstrates the highest affinity for secondary BAs [3, 35]. It is hence tempting to speculate that the intake of SPI with an HFD induces alterations in the ileal microbiota toward a configuration that tends to enhance the generation of secondary BAs, stimulating the secretion of GLP-1 by L-cells, which are most abundant in the ileum and colon. However, the dietary SPI did not affect blood glucose and insulin levels even it increased plasma GLP-1 levels. Further experiments would be required to characterize other physiological effects of the SPI.

Taken together, the results of this study demonstrate that the intake of SPI in an HFD exerts protective effects against HFD-induced weight gain and fat accumulation in multiple tissues by a mechanism that involves the gut microbiota-driven modulation of BA signaling and GLP-1 secretion in mouse models. Some clinical studies demonstrated the potential effects of soybean protein and its major component, β-conglycinin, on weight loss, reduction of serum triacylglycerol and visceral fat in pre-obese and obese subjects [18, 31]; although, the studies did not pursue the relationship of BAs and microbiome. Both primary and secondary BAs are reported as functional molecules through specific receptors, the bile acid composition to induce suitable activities still need to be elucidated in humans. Therefore, caution is necessary when extrapolating these results to human diets considering the substantial differences in the BA pool between species.

## Materials and methods

### Mice, diet and experimental design

Male C57BL/6 mice (7-week-old) were purchased from Japan SLC (Shizuoka, Japan) and maintained under a strict 12 h light/dark cycle and controlled atmospheric conditions (22°C, humidity) with free access to food and water. Mice were acclimated to the laboratory conditions on CLEA Rodent Diet (CE-2, CLEA Japan, Inc.) for 1 week. During the treatment, the 8-weeks-old mice were randomly assigned to high-fat diet (HFD) (n = 10) or SPI-containing HFD (HFD-SPI) (n = 10) for 4 weeks in a factorial design. The compositions of the diets are given in S1 Table. Body weight were measured once a week. Food intake was measured by every 2–3 days for 4 weeks and average of daily food intake (g/day/mouse) was calculated. Fecal droppings were collected at the end of treatment. All mice were then sacrificed under deep isoflurane-induced anesthesia. Liver, cecum (including the contents), epididymal, perirenal and subcutaneous adipose tissues were harvested and weighted. Blood was collected from the inferior vena cava using heparinized tubes and plasma was separated by immediate centrifugation (7,000 g, 5 min, 4°C). All tissues, feces and plasma were stored at −80°C until further processing. All experimental procedures involving mice were planned in accordance with the guidelines of the Committee on the Ethics of Animal Experiments of the Tokyo University of Agriculture and Technology and then these were approved by the Animal Research Ethics Subcommittee (permit number: 28–87). For HFD studies under sterile conditions, male germ-free ICR mice were housed in vinyl isolators under a 12-h light-dark cycle. 8-week-old germ-free ICR mice were placed on a HFD (50 kGy irradiated) or HFD-SPI (50 kGy irradiated) for 4 weeks (n = 8). All efforts were made to minimize suffering.

### Plasma biochemical analyses

Blood glucose was assessed using a portable glucometer with compatible glucose test strips (OneTouch ® Ultra ®, LifeScan, Milpitas, CA). Plasma cholesterol (LabAssay™ Cholesterol, Wako, Tokyo, Japan), NEFA (LabAssay™ NEFA, Wako), insulin [Insulin ELISA KIT (RTU), Shibayagi], PYY (Mouse/Rat PYY ELISA Kit, Wako) and triglyceride (LabAssay™ Triglyceride, Wako) levels were measured using commercial assay kits following manufacturer’s instructions. Plasma levels of GLP-1 were determined by enzyme-linked immunosorbent assay (ELISA) (GLP-1 (Active) ELISA KIT, Shibayagi, Gunma, Japan) following treatment with dipeptidyl peptidase IV (DPP-IV) inhibitor (Merck Millipore, Darmstadt, Germany), which prevents the degradation of active GLP-1.

### Hepatic histology

Livers were embedded in OCT compounds (SAKURA Finetek Japan) and sectioned at 7 μm. All slices were stained with hematoxylin and eosin (HE) for microscopic examination.

### Quantification of hepatic triacylglycerol contents

Hepatic triacylglycerol contents were measured according to the procedure described previously [23]. Briefly, liver homogenates were subjected to crude lipid extraction using the mixture of chloroform/methanol/0.5 M acetic acid. The organic phases were dried, and the sample was reconstituted in 2-propanol as assay samples. Triacyclglycerol levels were determined in the assay samples with a LabAssay™ Triglyceride kit.

### Quantification of short-chain fatty acids

For SCFAs measurement, SCFAs in feces were determined following a modified protocol as previously described [25].

### Quantification of bile acids

Cholic acid (CA), glyco-cholic acid (GCA), tauro-cholic acid (TCA), tauro-chenodeoxy cholic acid (TCDCA), α-muricholic acid (αMCA), β-muricholic acid (βMCA), Tauro-α-muricholic acid (TαMCA), Tauro-β-muricholic acid (TβMCA), deoxycholic acid (DCA), tauro-deoxycholic acid (TDCA), taurohyodeoxycholic acid (THDCA), tauro-ursodeoxycholic acid (TUDCA), lithocholic acid (LCA) and tauro-lithocholic acid (TLCA) were purchased from Rikaken (Tokyo, Japan).

Plasma (100 μL) was diluted with ultra-pure water (100 μL, Milli-Q [MQ] reference ultrapure water purification system; Merck Millipore, Darmstadt, Germany), alkalized with 0.4 M NaOH(aq) (200 μL), and the clarified by centrifugation (20,000 g, 15 min, 4°C). Clarified plasma was loaded onto an Oasis HLB μElution plate (Waters) that had been conditioned and equilibrated with methanol (200 μL) and ultra-pure water (400 μL), washed with ultra-pure water (100 μL), and eluted with methanol-acetonitrile (1:1, v/v, 100 μL) on an extraction plate vacuum manifold (Waters).

Lyophilized feces and cecal content (approx. 10 mg) were finely ground and well mixed with 0.2 M NaOH(aq) (1 mL). The mixtures were then purified from lipids using a liquid-liquid extraction with hexane (1 mL). The extraction step was repeated three times and the aqueous phases along with the solids were combined and incubated at 80°C for 20 min. After cooling to room temperature, the samples were centrifuged (20,000 g, 15 min, 4°C) and the supernatants were further cleaned up with Oasis PRiME HLB 1 cc cartridges (Waters) that had been conditioned with methanol (1 mL) followed by ultra-pure water (3 mL). The loaded cartridges were washed with ultra-pure water (500 μL) and the analytes were eluted with methanol-acetonitrile (1:1, v/v, 1 mL) for LC-MS analysis.

BAs were analyzed on an Acquity UPLC system coupled to a Waters Xevo TQD MS (Waters, Milford, MA, USA). The separation was achieved using an Acquity HSS C18 (2.1 3 100 mm, 1.7 mm) column (Waters) and gradient elution with 1% acetic acid aqueous solution and acetonitrile-isopropanol (9:1, v/v) as mobile phases. Analytes were detected by multiple reaction monitoring (MRM) in negative ion electrospray mode with source temperature and desolvation temperature set at 150 and 600°C, respectively. The MRM method parameters were determined by using IntelliStart™ (Waters). Analytes were quantified using external standards. Calibrators were prepared in methanol-acetonitrile (1:1, v/v) over the range of 0.001–1.0 μg/mL, with quality controls at 0.1 and 1.0 μg/mL.

### Analysis of fecal microbiota by 16S rRNA gene sequencing

Fecal DNA was extracted from frozen samples using the FastDNA® SPIN Kit for Feces (MP Biomedicals, Santa Ana, CA) per the manufacturer’s instructions. The V4 region of the 16S rRNA gene was amplified using dual-indexed 515F/806R primers. A KAPA Library Quantification kit for Illumina platforms (Kapa Biosystems, USA) was used to determine the library’s concentration and an Agilent Bioanalyzer high-sensitivity DNA analysis kit (Agilent, USA) was employed to determine the amplicon size. The amplicons were sequenced using an Illumina MiSeq with a MiSeq Reagent 222 kit V3 (Illumina, USA). The libraries were prepared following the Illumina protocol for 1 pM libraries: ‘Preparing Libraries for Sequencing on the MiSeq’ (part 15039740, Rev. D). Paired-end sequencing (2×301 bp) was carried out using Illumina MiSeq platform. Processing and quality filtering of reads were performed with Quantitative Insights into Microbial Ecology (QIIME) (v1.9.1). Paired reads were stitched with paired-end read merger (PEAR) and further filtered based on Phred quality scores (QN19) and for chimeric reads using USEARCH61. Filtered reads were demultiplexed within QIIME and samples with less than 5000 reads were excluded from further analysis. UCLUST was used to cluster sequences into operational taxonomical units (based on 97% identity). Operational taxonomic unit picking was performed using open-reference method, which encompasses clustering of reads against a reference sequence collection. To eliminate erroneous mislabeling, the resulting operational taxonomic unit tables were checked for mislabeling sequences. Representative sequences were further aligned using Python Nearest Alignment Space Termination with the SILVAcore-set alignment template. Construction of the phylogenetic tree was performed using the FastTree software method in QIIME. Sequence counts for each bacterial OTU and the phylogenetic tree were imported from Qiime into R (x64, 2.13.0) for principle component analysis (PCoA) with the R package Vegan 1.17–9 and for creating the heatmap of taxonomic relative abundance with the ComplexHeatmap package. LEfSe results were visualized using taxonomic histogram and cladogram, as implemented on http://huttenhower.sph.harvard.edu/galaxy/ with α = 0.05, the threshold of LDA score at 2.0 and relative abundance greater than 0.1%. The raw data have been deposited into the DNA Data Bank of Japan (DDBJ) database under the accession no. DRA007048.

For detection of Clostridium cluster XIVa, *Blautia coccoides* JCM1395T were provided from Japan Collection of Microorganisms of RIKEN BRC and used as standards specifically for the DNA-based determination of fecal bacterial counts. Bacterial DNA was isolated using MonoFas Bacterial Genomic Kit IV (GLC science, Tokyo, Japan) following the manufacturer’s instructions. Quantitative real-time-PCR analysis was performed with using SYBR Premix Ex Taq II (TaKaRa, Shiga, Japan) and the StepOnePlus™ Real-Time PCR System (Applied Biosystems, Foster City, CA). Bacterial primer sequences are as follows; Clostridium cluster XIVa, 5’-GGAGYATGTGGTTTAATTCGAAGCA-3’ (forward) and 5’-AGCTGACGACAACCATGCAC-3’ (reverse) [36].

### Statistical analysis

Statistical comparisons between the diet treatment groups were performed with two-tailed independent-samples t tests by assuming a normal distribution. The changes in the composition of the fecal microbiota were explored using the non-parametric Mann-Whitney U test at different taxon levels. Differences were considered significant for p-values <0.05. PERMANOVA test was conducted using the vegan package in R. All results are expressed as means ± SE. Statistically significant differences are shown with asterisks as follows: *, *p* < 0.05, **, *p* < 0.01.

## Supporting information

S1 Table Composition of HFD and SPI-containing HFD. These diets were prepared by research diet.
(DOC)

S1 Fig Body weight gain in the mice fed low fat diet or low-fat diet contained SPI. CONV-R mice were fed low fat diet (4% fat, NC) or low-fat diet contained SPI (NC_SPI) for 4 weeks. Data are expressed as means ± SE (n = 10 for each dietary group).
(TIF)
