# Supplementary material for: Dietary soybean protein ameliorates high-fat diet-induced obesity by modifying the gut microbiota-dependent biotransformation of bile acids
Source: PLoS One. 2018 Aug 13;13(8):e0202083. doi: 10.1371/journal.pone.0202083 (PMC6089412; doi:10.1371/journal.pone.0202083)
Supplement: S1 Table — These diets were prepared by research diet. (DOC) [file pone.0202083.s001.doc]

**S1 Table.** **Composition of HFD and SPI-containing HFD.**

| Ingredient | HFD | HFD-SPI^1^ |
| --- | --- | --- |
|  | g | g |
| Casein | 200 | 0 |
| Soy Protein Isolate | 0 | 203.3 |
| L-Cystine | 3 | 0 |
| DL-Methionine | 0 | 3 |
| Maltodextrin 10 | 125 | 125 |
| Sucrose | 68.8 | 68.8 |
| Cellulose, BW200 | 50 | 50 |
| Soybean oil | 25 | 25 |
| Lard | 245 | 245 |
| Mineral mix S10026^2^ | 10 | 10 |
| DiCalcium phosphate | 13 | 13 |
| Calcium carbonate | 5.5 | 5.5 |
| Potassium citrate, H_2_O | 16.5 | 16.5 |
| Vitamin mix V10001^3^ | 10 | 10 |
| Choline bitartrate | 2 | 2 |
| Total weight, g | 773.8 | 777.1 |
|  |  |  |
| Energy, kcal/g | 5.1 | 5.1 |
| % Energy |  |  |
| Protein | 18 | 18 |
| Carbohydrate | 21 | 21 |
| Fat | 61 | 61 |
| % Weight |  |  |
| Protein | 23 | 23 |
| Carbohydrate | 26 | 26 |
| Fat | 35 | 35 |

^1^Prepared by Research Diet, Inc., New Brunswick, NJ.

^2^The mineral mix composition was as follows (amount in 10 g): 0.5 g Mg, 0.33 g S, 1.0 g Na, 1.6 g Cl, 1.6 mg Mo, 2.0 mg Cr, 6.0 mg Cu, 0.2 mg I, 37.0 mg Fe, 59 mg Mn, 0.16 mg Se and 29 mg Zn.

^3^The vitamin mix composition was as follows (amount in 10 g): 4000 IU vitamin A palmitate, 1,000 IU vitamin D3, 50 IU vitamin E acetate, 0.5 mg menadione sodium bisulfite, 0.2 mg biotin, 10 μg cyanocobalamin, 2 mg folic acid, 30 mg nicotinic acid, 16 mg calcium pantothenate, 7 mg pyridoxine-HCl, 6 mg riboflavin and 6 mg thiamin HCl.
