# Supplementary figures and images for: Dietary soybean protein ameliorates high-fat diet-induced obesity by modifying the gut microbiota-dependent biotransformation of bile acids
Source: PLoS One. 2018 Aug 13;13(8):e0202083. doi: 10.1371/journal.pone.0202083 (PMC6089412; doi:10.1371/journal.pone.0202083)

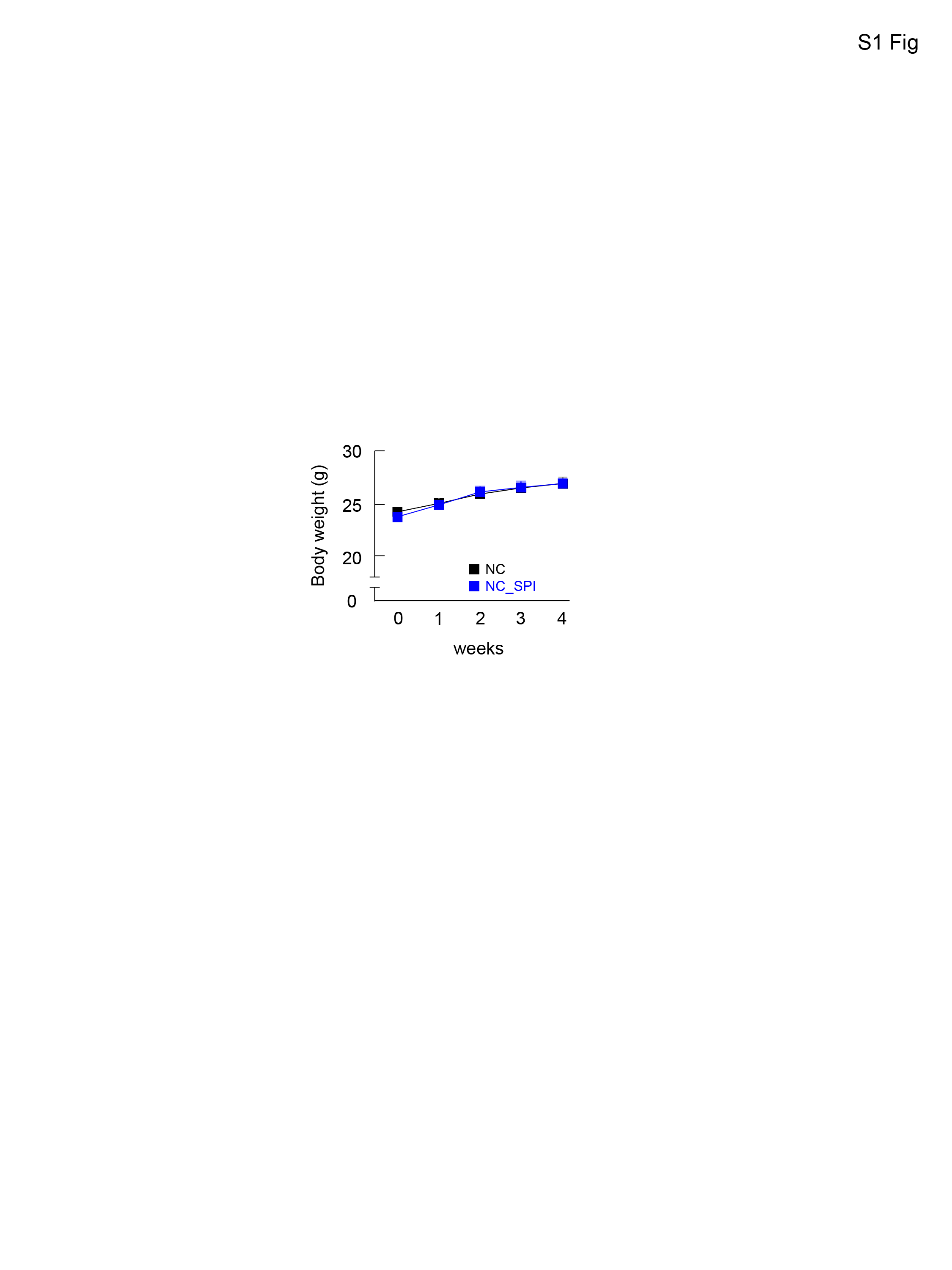

Supplement: S1 Fig — CONV-R mice were fed low fat diet (4% fat, NC) or low-fat diet contained SPI (NC_SPI) for 4 weeks. Data are expressed as means ± SE (n = 10 for each dietary group). (TIF) [file pone.0202083.s002.tif]
